# Palliative Care Training in Pediatric Nephrology Fellowship: A Cross-Sectional Survey

**DOI:** 10.34067/KID.0000000000000233

**Published:** 2023-08-02

**Authors:** Taylor R. House, Aaron Wightman, Jodi Smith, Margaret Schwarze, Miranda C. Bradford, Abby R. Rosenberg

**Affiliations:** 1 Department of Pediatrics, University of Wisconsin Madison, School of Medicine and Public Health, Madison, Wisconsin; 2 Department of Pediatrics, University of Washington, Seattle Children's Hospital, Seattle, Washington; 3 Department of Surgery and Department of Medical History and Bioethics, University of Wisconsin Madison, School of Medicine and Public Health, Madison, Wisconsin; 4 Biostatistics Epidemiology and Analytics in Research Core, Seattle Children's Research Institute, Seattle, Washington; 5 Dana-Farber Cancer Institute, Department of Psychosocial Oncology and Palliative Care, Boston, Massachusetts; 6 Boston Children's Hospital, Department of Pediatrics, Pediatric Advanced Care Program, Boston, Massachusetts; 7 Harvard Medical School, Department of Pediatrics, Division of Hematology-Oncology, Boston, Massachusetts

**Keywords:** children, patient-centered care, pediatric nephrology, pediatrics, quality of life

## Abstract

**Key Points:**

- Pediatric nephrology fellows have limited primary palliative exposure and opportunities to develop and refine primary palliative care (PC) skills.
- While experiential practice seems to improve confidence, most fellows have low confidence to provide primary PC.
- Fellows indicate a need and desire for additional PC training during nephrology fellowship.

**Background:**

Children with CKD and their families encounter significant burdens. Integrating primary palliative care (PC), holistic care provided by nephrologists focused on enhancing quality of life through symptom management, stress relief, and high-quality serious illness communication, provides an opportunity to promote flourishing. Incorporation of primary PC education in training is therefore recommended. Yet, adult nephrology fellows report inadequate preparation to deliver primary PC. Similar experience of pediatric nephrology fellows is unknown. We sought to describe pediatric nephrology fellows' experience in providing primary PC and PC exposure during training.

**Methods:**

We administered a cross-sectional web-based survey to pediatric nephrology fellows associated with the American Society of Pediatric Nephrology listserv in May 2021. The survey was adapted from a previously validated instrument and pretested by stakeholder nephrologists and subspecialty PC physicians; queries included institutional and personal PC experience, training, and confidence in primary PC delivery. Data were summarized descriptively.

**Results:**

Response rate was 32% (32/101). Respondents were 81% female and 50% White; 87% practiced in an urban setting. Only one fellow (3%) completed a PC rotation during fellowship, and 15 respondents (48%) completed a rotation in medical school or residency. Fellows reported substantially more practice conducting kidney biopsies than family meetings; 68% of fellows had performed >10 kidney biopsies, and 3% of fellows had led >10 family meetings. Confidence in navigating challenging communication, addressing psychological distress, or managing physical symptoms associated with CKD was generally low. Fellows with greater exposure to family meetings reported more confidence navigating challenging communication. Fellows endorsed a need for additional training; 97% indicated that training should happen during fellowship.

**Conclusions:**

Few pediatric nephrology fellows receive PC education or exposure during training, resulting in low rates of knowledge and confidence in primary PC delivery. Fellows indicate a need and desire for improved PC training.

## Graphical abstract

**Figure:**
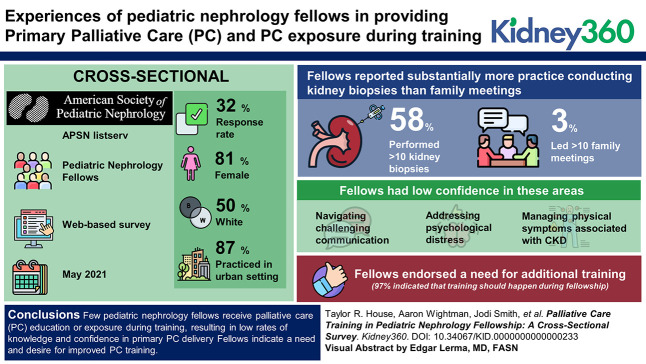

## Introduction

Children with CKD and their families face enormous burdens associated with their illness. Compared with healthy peers, they face earlier death. In studies of healthy controls and children with other chronic illnesses, children with CKD experience worse health-related quality of life.^1–9^ Physical and psychological symptoms are highly prevalent among children with CKD.^10–14^ Up to 60% of children on dialysis report moderate-to-severe pain in their daily lives, and in studies across stages of CKD, about half of children report pain interfering with their daily activities.^9,15^ Variable, concurrent physical symptoms include edema, gastrointestinal distress, fatigue, sleep disturbances, and pruritis.^16^ Up to half of this population suffer from poor mental health.^11,12^ Children with CKD also experience difficulty in engaging in decision-making with their parents and nephrologists.^7^ These burdens extend to siblings and caregivers and evolve after kidney transplant.^17–23^ Integration of primary palliative care (PC) within nephrology care offers an opportunity to alleviate these burdens of children with CKD and their families.^24^

Primary PC, holistic care delivery focused on optimizing quality of life, is critical for any clinician serving patients with serious illness.^25^ The benefit of primary PC has been well-established in comparable pediatric chronic disease states including cancer, cystic fibrosis, and congenital heart disease.^26–30^ Furthermore, PC branded as kidney supportive care has been recognized as a vital component of adult nephrology care with calls for its inclusion among all patients with advanced CKD.^31^ Subsequently, focused efforts in research, education, and clinical care have emerged.^32^ However, despite a demonstrable need and desire for PC-focused training, adult nephrology fellows continue to report feeling ill-prepared to provide primary PC.^33^ Pediatric nephrology fellows' perspectives of PC are unknown. We sought to describe pediatric nephrology fellows' experience in providing primary PC and PC exposure during training.

## Methods

### Study Design

We performed an observational, anonymous, cross-sectional web-based survey study of pediatric nephrology fellows in the United States. We electronically distributed the survey to eligible participants—pediatric nephrology fellows who subscribe to the American Society of Pediatric Nephrology (ASPN) listserv and practice in the United States—in May 2021. To ensure proper sampling, at the start of the survey, we asked participants to respond to two mandatory questions identifying themselves as trainee pediatric nephrologists currently practicing in the United States. If these inclusion criteria were unmet, the survey closed automatically. Given the total number of current fellows participating in the ASPN listserv is unknown, we used the prior 3 years Accreditation Council for Medical Education (ACGME) match data to estimate the total number of current pediatric nephrology fellows in the United States.^34–36^ We sent an initial email introducing the study and survey followed by reminder emails to participate 2 and 3 weeks after initial contact. After survey completion, participants could elect to enter a raffle for a $100 gift card as compensation for their time, which was recorded separately from survey responses. The study was determined to be exempt from review by the Seattle Children's Hospital Institutional Review Board.

### Survey Development

The survey was adapted from the Provider Survey about PC for Children with Heart Disease, a previously validated survey tool used to investigate pediatric cardiology physicians' attitudes about PC.^37,38^ The survey primarily included close-ended questions with multiple-choice and five-point Likert response options. Initially, we reviewed the pertinent PC and nephrology literature to modify the survey for pediatric nephrology fellows. We next convened a panel of two pediatric nephrologists, three pediatric subspecialty PC physicians, and two pediatric nephrology fellows to review survey content and design. We then pilot tested the survey with stakeholder physicians, performed debriefings, made modifications, and established the final version.^39^ Final domains included institutional and personal experience, training and education, physician confidence with primary and subspecialty PC delivery, and opinions regarding clinical team roles and responsibilities. We specifically queried (*1*) prior participation in PC rotations during medical training (multiple-choice); (*2*) knowledge of PC principles (five-point Likert scale with options ranging from no=1 to extensive=5 knowledge and a multiple-choice question probing means of PC knowledge acquisition); (*3*) fellows' experiences performing kidney biopsies and conducting family meetings (multiple-choice options of never, 1–2, 3–6, 7–10, and >10 times based on a previously validated survey instrument performed in adult nephrology fellows)^33^; (*4*) aspects of local PC resources including barriers, timing, and criteria for automatic subspecialty PC consultation (multiple-choice and timing options including too early, at the appropriate time, too late, and contact too limited to assess); (*5*) perspectives on the indications, role, and involvement of subspecialty PC clinicians and resources generally and among children with various nephrology diagnoses and needs (multiple-choice, five-point Likert scale with options ranging from strongly disagree to strongly agree and never to always); (*6*) fellows' confidence addressing needs across PC domains including physical symptoms, psychosocial well-being, and challenging communication (five-point Likert scale with options ranging from not at all to very confident); (*7*) fellows' opinions regarding which clinicians should be responsible for leading goals-of-care conversations for nephrology patients across care settings (multiple-choice options of primary pediatric nephrologist, primary care clinician, PC physician, transplant surgeon, intensivist, hospitalist, other or determined on a case-by-case basis); and (*8*) areas of primary PC delivery in which fellows could benefit from additional training, preferred formats for educational opportunities, and ideal timing for PC training (multiple-choice selecting all that apply, five-point Likert scale with options ranging from very unlikely to very likely in questions of participation in proposed PC learning opportunities). Finally, we obtained demographic information including years since medical school graduation, sex, race, ethnicity, hospital size, practice geographic location, and kidney replacement offerings at their institution.

### Data Management and Statistics

Study data were collected and managed using REDCap electronic data capture tools hosted at Seattle Children's Hospital.^40,41^ Responses were summarized descriptively using counts, percentages, and graphical representations. Analyses were performed using Stata version 16 (StataCorp, College Station, TX). For questions of physician confidence, responses were dichotomized as low confidence (not at all, a little, or somewhat response selections) vs high confidence (moderately or very response selections). In questions of likelihood of participation in future training activities, responses were similarly dichotomized.

## Results

### Characteristics of Respondents

A total of 32 fellows participated, with a response rate of 32% (32/101). Participants were not required to answer every question, so some items had response totals <32. Participants were 81% female and 50% White, with 63% practicing in hospitals with over 200 pediatric beds and 84% working in hospitals featuring subspecialty PC teams (Table 1). Geographic location of fellows' programs varied with 50% in the Northeast, 13% in the South, 10% in the Midwest, and 27% in the West and the majority (87%) located in an urban area. All fellows' institutions offered continuous kidney replacement therapy, in-center pediatric hemodialysis, and peritoneal dialysis. A plurality of fellows (40%) was exposed to an average of 10–20 kidney transplants annually, and 19% performed prenatal counseling as an aspect of their clinical practice.

**Table 1:** Participant Characteristics

Characteristic	Total (*N*=32)^a^
**Sex, *n* (%)**	
Female	26 (81)
Male	6 (19)
**Race, *n* (%)**	
Asian	9 (28)
Black	2 (6)
Native Hawaiian or Pacific Islander	1 (3)
Multiracial other	4 (13)
White	16 (50)
**Ethnicity, *n* (%)**	
Hispanic or Latino	5 (16)
**Fellowship location, *n* (%)**	
Northeast	15 (50)
South	4 (13)
Midwest	3 (10)
West	8 (27)
**Inpatient pediatric beds, *n* (%)**	
<50 beds	1 (3)
50–100 beds	7 (23)
101–200 beds	3 (10)
>200 beds	19 (63)
Access to palliative care team, *n* (%)	27 (84)

a Participants were not required to answer every question; responses may not total 32.

### Palliative Care Education and Experience

About half of fellows (48%) completed a PC rotation during medical school or residency, and only one fellow (3%) completed a PC rotation during fellowship. Fellows reported a mean knowledge of PC principles of 2.3±1. For fellows who described having knowledge of PC principles, the most common means of acquiring knowledge was through experience with subspecialty PC consultation for their patients (61%) or through didactic sessions in training (45%). Eight fellows (26%) reported having no PC knowledge. Only two fellows (6%) reported receiving training in caring for children with CKD at the end of life.

**Figure 1:**
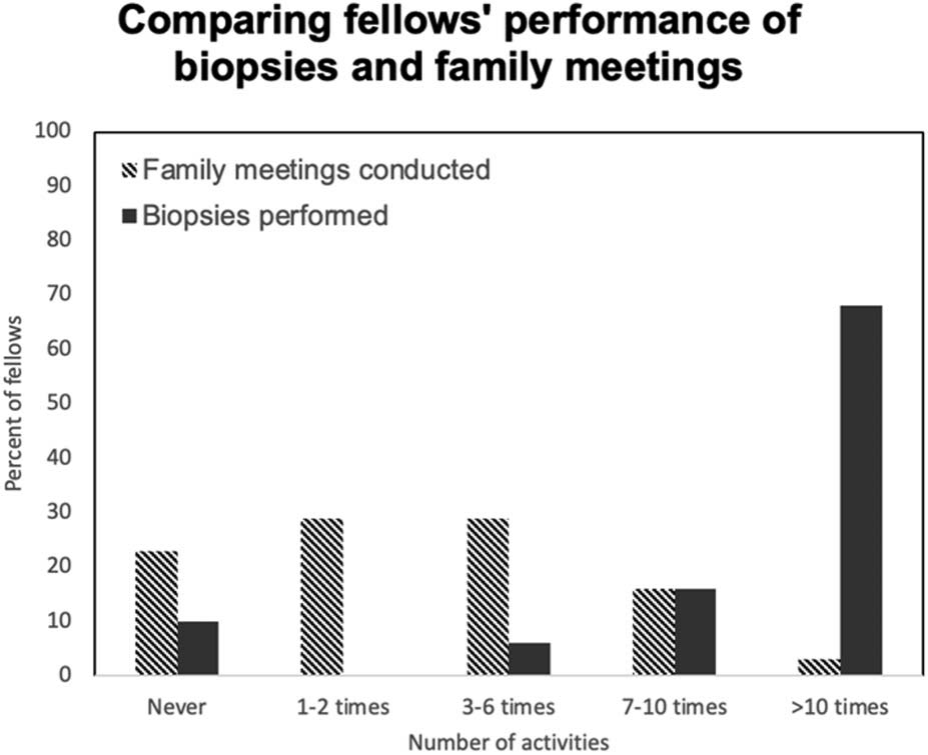
Comparison of fellow participation in kidney biopsies versus family meetings.

As compared with performing kidney biopsies, fellows reported far fewer opportunities to lead family meetings (Figure 1). Sixty-eight percent had performed over 10 kidney biopsies, and 10% had never performed a kidney biopsy. By contrast, 3% of fellows had led over 10 family meetings, and 23% had never led such a meeting.

**Figure 2:**
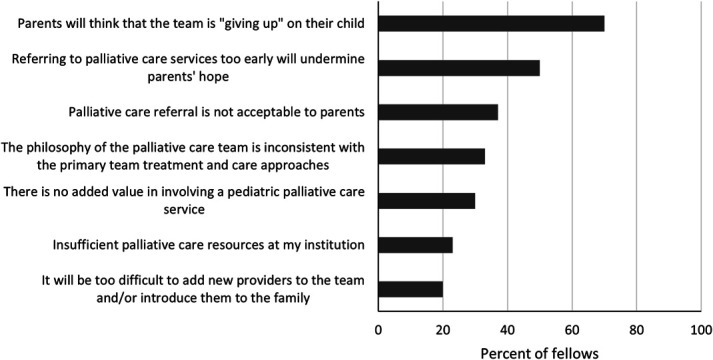
Fellows' perceived barriers to palliative care consults for children with CKD.

Nearly all fellows (94%) found prior subspecialty PC consultations to be helpful, and 48% reported few or no barriers to requesting a PC consult at their institution. When asked about their perceptions of PC involvement, 87% and 90% indicated that they either disagreed or strongly disagreed that PC and kidney transplant or maintenance dialysis are mutually exclusive, respectively; 77% agreed or strongly agreed that children on dialysis should receive outpatient PC resources. In addition, 60% indicated that subspecialty PC consultation should happen at the time of diagnosis for life-threatening conditions for which cure is feasible but may fail. Yet, PC consults were perceived as happening too late nearly two-thirds of the time (65%), and PC resources were infrequently used for patients with CKD. Only one fellow (3%) cared for three or more patients on chronic dialysis who received subspecialty PC resources. The most cited barrier to involvement of the PC team for children with CKD was the concern that parents will think that the team is giving up on their child (68%), followed by the belief that referring to PC services too early will undermine parents' hope (48%; Figure 2).

### Perceived Physician Confidence

**Figure 3:**
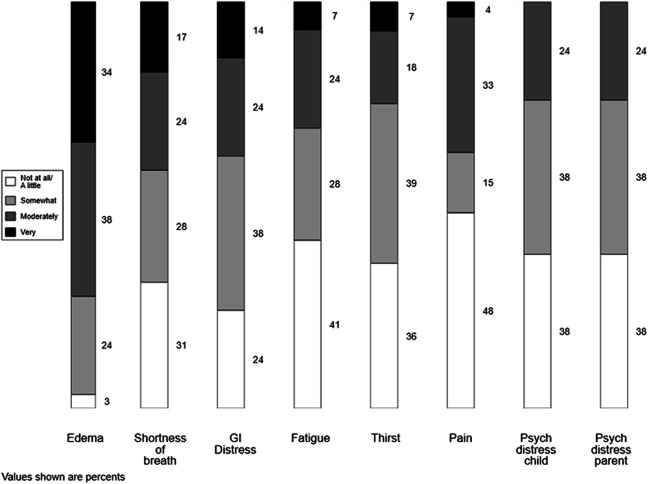
**Perceived fellow confidence in managing symptoms for children with CKD.** Number of responding fellows was 29 except in regard to thirst (*n*=28) and pain (*n*=27).

Across domains of primary PC including physical symptom management, psychosocial well-being, and navigating challenging communication, fellows reported low confidence levels. Fewer than half of fellows felt very or moderately confident to manage pain, shortness of breath, gastrointestinal distress, fatigue, or thirst of a child with CKD (Figure 3). Of symptoms queried, edema was the single physical symptom fellows felt confident managing, with 72% feeling moderately or very confident. No fellow indicated that they felt very confident managing psychological distress of a child with CKD or their parent; less than a quarter (24%) felt moderately confident to do so. About two-thirds (69%) felt low confidence providing care to a child with CKD at the end of life.

Fellows also reported low confidence navigating challenging communication. Only 31%, 28%, and 17% of fellows reported confidence discussing goals of care, code status, or prognosticating life expectancy for a child with CKD, respectively. Despite not feeling confident, most fellows felt responsible to perform these tasks. When asked to consider which clinician should be responsible to discuss limitations of life-prolonging interventions for nephrology patients, 84% of fellows indicated that the nephrologist should perform this duty in the outpatient setting, and 61% specified that the nephrologist should do so in an inpatient setting.

While the sample size precludes association testing, fellows with increased exposure to family meetings also reported greater confidence navigating challenging communication. For example, the proportion who were moderately or very confident discussing goals of care, code status, or prognosticating life expectancy for a child with CKD was 71%, 86%, and 71%, respectively, among respondents who had led at least seven family meetings (*n*=7), compared with 23%, 9%, and 0% among those who had led fewer than seven (*n*=22). By contrast, participation in a PC rotation at any point during medical training was not associated with substantially higher confidence. The proportion of fellows who were moderately or very confident discussing goals of care, code status, or prognosticating life expectancy was 31%, 31%, and 19% among respondents who had participated in a rotation (*n*=16), compared with 31%, 23%, and 15% among those who had not (*n*=13).

### Perspectives on Future Training

**Figure 4:**
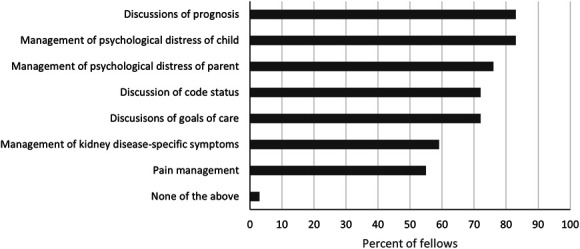
Palliative care content areas in which fellows indicate desire for more training.

Fellows indicated a willingness and desire for additional PC training (Figure 4). Further training in navigating challenging communication was of particular interest, with 83% desiring more training in prognosis discussions, 72% in goals of care discussions, and 72% in code status discussions. Reflecting the lack of confidence in managing psychological distress, 83% and 76% of fellows wanted additional training in managing psychological distress of a child with CKD or their caregiver, respectively. Over half of fellows (59%) also desired more training in managing symptoms related to CKD. While several fellows indicated that incorporating additional PC training is appropriate during residency (55%) or as an attending (52%), fellows predominantly expressed that additional training should happen during nephrology fellowship (97%).

## Discussion

In this national sample of pediatric nephrology fellows, we found that fellows encounter limited PC exposure and opportunities for practice. Fellows identify that PC skills and resources are beneficial in the care of children with CKD, and they have low confidence to attend to PC-focused needs. Subsequently, most fellows indicate a desire for additional PC training during nephrology fellowship.

Fellows' primary source of PC exposure was through consultation with PC specialists, and as compared with traditional nephrology procedures such as kidney biopsies, fellows led family meetings infrequently. These findings mirror those among adult nephrology fellows.^33^ This is important because communication (including leading family meetings and sharing serious news) is an evidence-based procedure with similar rigor and steps as a kidney biopsy.^42^ Despite reporting few opportunities and low confidence to engage in challenging communication, fellows felt a responsibility to do so. The majority specified that nephrologists should be the primary clinician to discuss limitations of life-prolonging therapies with patients and families, and almost all suggested this type of procedural training occur during fellowship. These opinions align with guidance from the Renal Physicians Association Clinical Practice Guideline on Shared Decision-Making and have implications for pediatric nephrology fellowship directors and training programs.^43^ It is vital to support developing pediatric nephrologists in meeting their endorsed responsibilities in providing evidence-based supportive communication. This gap could be addressed through adaptation and incorporation of existing communication curriculum such as NephroTalk, an educational program designed to equip nephrologists with primary PC communication skills.^44,45^

Our findings also suggest that experience promotes confidence; fellows who reported more exposure to family meetings also reported more confidence navigating challenging communication. By contrast, a similar finding was not seen among fellows with increased exposure to PC rotations. This could suggest that while PC education during medical school or residency is helpful, there is specific value in providing experiential primary PC education during fellowship. Indeed, much of medical education relies on see one, do one practice-based learning. Medical students and residents tend to observe, rather than lead family meetings. Alternatively, fellows may develop confidence by leading such discussions, and it may be most impactful to receive this practice during nephrology fellowship while concurrently learning to holistically care for children with CKD and their families. This approach leverages successful adult learning principles.^44,46^

Fellows generally demonstrated low confidence to manage physical symptoms and psychosocial distress that may accompany a child and family's CKD experience. This is concerning because physical and psychological symptoms are highly prevalent among children with CKD. Furthermore, increasingly severe symptoms are associated with poorer quality of life and health outcomes including adherence.^8,9,15,47^ Thus, it is imperative that pediatric nephrologists be equipped to address these symptoms. Formal training on symptom management should be included in future PC curriculum for pediatric nephrology fellows.

Participants demonstrated a positive perception of prior subspecialty PC consults and the potential benefits of leveraging PC resources for their patients. However, PC resources were infrequently applied for patients of responding fellows. A possible explanation for underutilization is the persistent stigma of PC and its false association with end-of-life care or hospice. Fellows indicated concern that PC referral could signify giving up or diminishing hope. While addressing this stigma through rebranding as in adult nephrology is a consideration, this also highlights a unique opportunity to bolster the primary PC skills of pediatric nephrologists and promote utilization of pediatric nephrologists with additional PC skillsets.^24,31^ Patients have PC needs that require care, yet pediatric nephrologists may not be equipped to meet these needs, and it is impractical to consider subspecialty PC consultation for each child with CKD. Using the expertise of a pediatric nephrologist with additional PC training could bridge this gap, meeting patients' needs and serving as a funnel into subspecialty PC consultation for complex cases. Incorporating this care as a routine component of nephrology helps alleviate the stigma of PC.^24^

Importantly, respondents' enthusiasm for the benefits of PC in the care of children with CKD mirrors evolutions in pediatric nephrology and PC. Within nephrology, scientific advances have allowed for a shift in care focus to highlight the importance of living well in addition to living longer. While subspecialty pediatric PC is a relatively young field, it has similarly advanced from a narrow view of care provided at the end of life to a broader scope emphasizing comprehensive care through disease stages pursued concurrent with curative treatments.^48^ Taken together, there is an increasing recognition of and demand for PC integration within pediatric nephrology corroborated by fellows' perspectives in our findings.

This study has several important limitations. While this is the first inquiry into PC experience of pediatric nephrology fellows, and response rate is consistent with findings in other web-based physician surveys, our low response rate may limit generalizability.^49^ Fellows who responded may have more (or less) experience with or interest in PC than nonrespondents. Because the number of fellows participating in the listserv was not available requiring use of ACGME data to determine the total number of active pediatric nephrology fellows in the country, it is also possible that our calculated response rate underestimates the actual response rate. Our sample consisted predominantly of White, female fellows training at large, urban centers, and perspectives from diverse clinicians from smaller centers may be lacking. This is of particular interest because smaller centers may have lower access to subspecialty PC services.^50^ In addition, responses were based on fellow report rather than direct observation introducing potential for self-report bias.

Nevertheless, this study is the first to investigate the perspectives of pediatric nephrology fellows regarding experience, education, and confidence with PC. Fellows reported limited opportunities to engage in PC skill development and education, resulting in low knowledge and confidence across care domains. They were open to utilization of PC services for their patients, and concerns about family perceptions or ability to successfully deliver PC were barriers. There was a demonstrated need and desire for PC training and education during pediatric nephrology fellowship. Future work should focus on garnering additional stakeholder input in delivery of PC to children with CKD and development and implementation of primary PC curriculum for pediatric nephrology fellows that maximizes opportunities to practice challenging communication and symptom management. Enhancing the primary PC skills of pediatric nephrologists offers the chance to improve the care of children with CKD and their families.

## Data Sharing Statement

All data are included in the manuscript and/or supporting information.
